# Selecting Classification Methods for Small Samples of Next-Generation Sequencing Data

**DOI:** 10.3389/fgene.2021.642227

**Published:** 2021-03-04

**Authors:** Jiadi Zhu, Ziyang Yuan, Lianjie Shu, Wenhui Liao, Mingtao Zhao, Yan Zhou

**Affiliations:** ^1^Department of Mathematics and Statistics, Xidian University, Xi'an, China; ^2^Shenzhen Key Laboratory of Advanced Machine Learning and Applications, College of Mathematics and Statistics, Institute of Statistical Sciences, Shenzhen University, Shenzhen, China; ^3^Faculty of Business Administration, University of Macau, Macau, China; ^4^GuangDong University of Finance, Guangzhou, China; ^5^Institute of Statistics and Applied Mathematics, Anhui University of Finance and Economics, Bengbu, China

**Keywords:** RNA-seq data, classification, PLDA, NBLDA, ZIPLDA, ZINBLDA

## Abstract

Next-generation sequencing has emerged as an essential technology for the quantitative analysis of gene expression. In medical research, RNA sequencing (RNA-seq) data are commonly used to identify which type of disease a patient has. Because of the discrete nature of RNA-seq data, the existing statistical methods that have been developed for microarray data cannot be directly applied to RNA-seq data. Existing statistical methods usually model RNA-seq data by a discrete distribution, such as the Poisson, the negative binomial, or the mixture distribution with a point mass at zero and a Poisson distribution to further allow for data with an excess of zeros. Consequently, analytic tools corresponding to the above three discrete distributions have been developed: Poisson linear discriminant analysis (PLDA), negative binomial linear discriminant analysis (NBLDA), and zero-inflated Poisson logistic discriminant analysis (ZIPLDA). However, it is unclear what the real distributions would be for these classifications when applied to a new and real dataset. Considering that count datasets are frequently characterized by excess zeros and overdispersion, this paper extends the existing distribution to a mixture distribution with a point mass at zero and a negative binomial distribution and proposes a zero-inflated negative binomial logistic discriminant analysis (ZINBLDA) for classification. More importantly, we compare the above four classification methods from the perspective of model parameters, as an understanding of parameters is necessary for selecting the optimal method for RNA-seq data. Furthermore, we determine that the above four methods could transform into each other in some cases. Using simulation studies, we compare and evaluate the performance of these classification methods in a wide range of settings, and we also present a decision tree model created to help us select the optimal classifier for a new RNA-seq dataset. The results of the two real datasets coincide with the theory and simulation analysis results. The methods used in this work are implemented in the open-scource R scripts, with a source code freely available at https://github.com/FocusPaka/ZINBLDA.

## 1. Introduction

RNA sequencing (RNA-seq), which involves directly sequencing complementary DNAs and aligning the sequences to the reference genome or transcriptome, has emerged as a powerful technology for measuring gene expression (Mardis, 2008; Morozova et al., 2009; Wang et al., 2009). In recent years, the affordability and effectiveness of RNA-seq has resulted in its application in biological and medical studies, such as genomics research (Nagalakshmi et al., 2008; Trapnell et al., 2010) and clinical use (Berger et al., 2010; Biesecker et al., 2012). Unlike microarray technology, RNA-seq allows for the detection of novel transcripts with low background signals. One of the biological applications of RNA-seq is inferring differential expression (DE) genes between different conditions or tissues. Existing popular methods include edgeR (Robinson and Smyth, 2008; Robinson et al., 2010), DESeq2 (Love et al., 2014), and LFCseq (Lin et al., 2014). Another important application is the diagnosis of diseases. Numerous discriminant methods have been proposed for the diagnosis of diseases using microarray data, such as diagonal linear discriminant analysis and diagonal quadratic discriminant analysis in Dudoit et al. (2002). In previous RNA-seq experiments, the read counts (the number of short reads mapped to the reference genome) have been used to measure the expression level. However, because the expression matrix entries are non-negative integers, classification methods that follow a Gaussian distribution may not perform well for RNA-seq data.

Classification methods based on different discrete distributions have been proposed for RNA-seq data. Witten (2011) assumed RNA-seq data follow a Poisson distribution and proposed a Poisson linear discriminant analysis (PLDA) method. Comparison studies (Tan et al., 2014) have shown that PLDA performs much better than the method used for microarray data when classifying RNA-seq data. Considering the overdispersion of RNA-seq data, Dong et al. (2016) assumed that data follow a negative binomial distribution and developed a negative binomial linear discriminant analysis (NBLDA) method. Zhou et al. (2018) found excess zeros in real RNA-seq data and proposed a zero-inflated Poisson logistic discriminant analysis (ZIPLDA) method, which assumes RNA-seq data follow a mixture distribution with a point mass at zero and a Poisson distribution.

Due to the shallow sequence depth and dispersed biological replicates, there may be excess zeros and overdispersion in a real RNA-seq dataset, which should be considered when conducting data analysis. For instance, the real dataset TCGA-LIHC, which includes a cancerous and normal group, contains about 43.24% zeros of all numerical values, and the estimated dispersion parameter is 1.12. Therefore, a natural assumption would be to extend the existing discrete distribution to a mixture distribution with a point mass at zero and a negative binomial distribution. We call this method zero-inflated negative binomial logistic discriminant analysis (ZINBLDA). To obtain the model, which is similar to ZIPLDA, we built a mixture distribution with a point mass at zero and a negative binomial distribution for the remaining data. We then estimated the parameters in the model. Finally, we obtained a classifier by Bayes rule to predict for a future observation. We also analyzed the relationship between the above four classification methods, and the resulting discriminant scores for the four classification methods showed that they can transform into each other in some cases. We examined these four methods from the perspective of their parameters and determined how the parameters provide the link between the selected optimal method and the model classification performance. In addition, we built a decision tree to help us select the optimal classifier from these four methods for a new dataset.

The remainder of the article is organized as follows. In section 2, we review the existing three classification methods and propose the ZINBLDA method for overdispersion RNA-seq data with an excess of zeros. We also give the estimation of the parameters in the model in detail. We further discuss the transformation relations between the four methods. Section 3 discusses the results of the simulation studies that were conducted to evaluate the performance of the four methods in a wide range of settings. This section also presents a decision tree that was built to select the optimal classifier from these four methods for a new dataset. In section 4, we employ the four methods to analyze two real RNA-seq datasets and evaluate their performance. Finally, we conclude the work with a discussion of the findings and future directions.

## 2. Classification Methods

There are three existing classification methods for RNA-seq data: PLDA (Witten, 2011), NBLDA (Dong et al., 2016), and ZIPLDA (Zhou et al., 2018). We propose a new discriminant analysis method to model overdispersion RNA-seq data with excess zeros. We examined these four methods from the perspective of their parameters and analyzed the transformation relations between the methods.

Before introducing the methods, we must first specify some notations used in this work. In this paper, *K* is the number of classes, and *X*_*ki*_*k*_*g*_ denotes the number of read counts that are mapped to gene *g* in sample *i*_*k*_ of class *k*, where *k* = 1, ⋯, *K*; *i*_*k*_ = 1, ⋯, *n*_*k*_; and *g* = 1, ⋯, *G*. Specifically, there are *n*_*k*_ samples in class *k*, and $n=\overset{K}{\underset{k=1}{\sum }}n_{k}$ denotes the total number of samples for all classes.

### 2.1. Principle of the Classifiers

The principle of the classifiers is applicable for the following four classifiers. Suppose that for the training set ${\left\{\left(x_{i},y_{i}\right)\right\}}_{i=1}^{n}$ we wished to classify a new observation $x^{*}\text{​​}=\text{​​ }{\left(X_{1}^{*},\cdots ,X_{G}^{*}\right)}^{T}$. If y^*^ is the unknown label of x^*^, by Bayes' rule

(1) $$\begin{array}{l} P\left(y^{*}=k|x^{*}\right)\propto f_{k}\left(x^{*}\right)\pi_{k}, \end{array}$$

where *f*_*k*_ is the probability density function of an observation in class *k*, and π_*k*_ is the prior probability that an observation belongs to class *k*. In general, we can use π_*k*_ = *n*_*k*_/*n* to satisfy $\overset{K}{\underset{k=1}{\sum }}\pi_{k}=1$. We define a discriminant score function as $d_{k}\left(x^{*}\right)=\log\left[P\left(x^{*}|y^{*}=k\right)\pi_{k}\right]$ on the basis of formula (1) and assign a new observation to the class for which the discriminant score is the highest.

### 2.2. Poisson Linear Discriminant Analysis

For PLDA, Witten (2011) assumed that RNA-seq data follow a Poisson distribution, that is,

(2) $$\begin{array}{l} X_{ki_{k}g}|y_{i_{k}}=k\sim Poisson\left(\mu_{ki_{k}g}\right),\;\mu_{ki_{k}g}=d_{kg}s_{i_{k}}\lambda_{g}, \end{array}$$

where μ_*ki*_*k*_*g*_ is the expectation for gene *g* in sample *i*_*k*_ of class *k*, *s*_*i*_*k*__ is the size factor used to identify individuals in the *k*th class, λ_*g*_ is the total number of read counts for gene *g*, and *d*_*kg*_ allows for the differential expression of gene *g* between the different classes. Following the expression of (2), the probability density function is

$$\begin{array}{l} P\left(X_{ki_{k}g}=x_{ki_{k}g}\right)=\frac{\mu_{ki_{k}g}^{x_{ki_{k}g}}}{\left(x_{ki_{k}g}\right)!}e^{-\mu_{ki_{k}g}}. \end{array}$$

Thus, according to formula (1), the discriminant score of PLDA is obtained by

(3) $$\begin{array}{l} d_{k}\left(x^{*}\right)=\overset{G}{\underset{g=1}{\sum }}X_{g}^{*}\log\left(d_{kg}\right)-s^{*}\overset{G}{\underset{g=1}{\sum }}\lambda_{g}d_{kg}+\log\pi_{k}+C, \end{array}$$

where s^*^ is the size factor of test observation, and *C* represents a constant that is unrelated to the class label.

### 2.3. Negative Binomial Linear Discriminant Analysis

Modeling RNA-seq data with a negative binomial distribution instead of a Poisson distribution is a natural extension. Dong et al. (2016) proposed NBLDA to allow for cases where variance is greater than or equal to the mean, and they also demonstrated that NBLDA is more suitable when biological replicates are available. The negative binomial distribution is expressed as

(4) $$\begin{array}{l} X_{ki_{k}g}|y_{i_{k}}=k\sim NB\left(\mu_{ki_{k}g},\phi_{g}\right),\;\mu_{ki_{k}g}=d_{kg}s_{i_{k}}\lambda_{g}, \end{array}$$

where ϕ_*g*_ is a non-negative dispersion parameter, and the rest of parameters are the same as for PLDA. Therefore, the probability density function of *X*_*ki*_*k*_*g*_ = *x*_*ki*_*k*_*g*_ in model (4) is

$$\begin{array}{l} \begin{array}{cc} \;P\left(X_{ki_{k}g}=x_{ki_{k}g}\right) & =\frac{\Gamma \left(x_{ki_{k}g}+\phi_{g}^{-1}\right)}{\left(x_{ki_{k}g}\right)!\Gamma \left(\phi_{g}^{-1}\right)}{\left(\frac{\mu_{ki_{k}g}\phi_{g}}{1+\mu_{ki_{k}g}\phi_{g}}\right)}^{x_{ki_{k}g}}\\ (\frac{1}{1+\mu_{ki_{k}g}\phi_{g}}{)}^{\phi_{g}^{-1}}. \end{array} \end{array}$$

Similarly, the discriminant score can be obtained by

(5) $$\begin{array}{l} d_{k}(x^{*})=\sum_{g=1}^{G}X_{g}^{*}[\log(d_{kg})-\log(1+s^{*}\lambda_{g}d_{kg}\phi_{g})]\\ \;-\sum_{g=1}^{G}\phi_{g}^{-1}\log(1+s^{*}\lambda_{g}d_{kg}\phi_{g})+\log\pi_{k}+C. \end{array}$$

### 2.4. Zero-Inflated Poisson Logistic Discriminant Analysis

Considering data with excess zeros due to missing records or no observation signal, Zhou et al. (2018) proposed ZIPLDA method, which assumes that data follow a zero-inflated Poisson distribution. The distribution is expressed as

$$X_{ki_{k}g}\;\sim \;\left\{ \begin{array}{l} \delta_{\left\{0\right\}},\;p_{ki_{k}g},\\ \text{Poisson}(\mu_{ki_{k}g}),\;(1-p_{ki_{k}g}), \end{array}\right.$$

where δ_{0}_ denotes the point mass at zero, *p*_*ki*_*k*_*g*_ is the probability of δ_{0}_ in gene *g* of sample *i*_*k*_ in class *k*, and μ_*ki*_*k*_*g*_ is same as in the former two classifiers. Thus, the probability of *X*_*ki*_*k*_*g*_ is written as

$$P(X_{ki_{k}g})=\left\{ \begin{array}{l} p_{ki_{k}g}+(1-p_{ki_{k}g})e^{-\mu_{ki_{k}g}},\;X_{ki_{k}g}=0,\\ \;(1-p_{ki_{k}g})\frac{\mu_{ki_{k}g}^{X_{ki_{k}g}}}{(X_{ki_{k}g})!}e^{-\mu_{ki_{k}g}},\;X_{ki_{k}g}>0. \end{array}\right.$$

Additionally, the probability density function of *X*_*ki*_*k*_*g*_ = *x*_*ki*_*k*_*g*_ is

$$\begin{array}{l} P(X_{ki_{k}g}=x_{ki_{k}g})={\left[p_{ki_{k}g}+(1-p_{ki_{k}g})e^{-\mu_{ki_{k}g}}\right]}^{I_{\left(x_{ki_{k}g}=0\right)}}\\ \;{\left[(1-p_{ki_{k}g})\frac{\mu_{ki_{k}g}^{x_{ki_{k}g}}}{(x_{ki_{k}g})!}e^{-\mu_{ki_{k}g}}\right]}^{I_{\left(x_{ki_{k}g}>0\right)}}. \end{array}$$

Finally, the discriminant score $d_{k}\left(x^{*}\right)$ is,

(6) $$\begin{array}{l} d_{k}(x^{*})=\sum_{g=1}^{G}I_{\left(X_{g}^{*}=0\right)}\log\left({\hat{p}}_{kg}^{*}+(1-{\hat{p}}_{kg}^{*})e^{-d_{kg}s^{*}\lambda_{g}}\right)\\ \;-\sum_{g=1}^{G}I_{\left(X_{g}^{*}>0\right)}d_{kg}s^{*}\lambda_{g}+\sum_{g=1}^{G}I_{\left(X_{g}^{*}>0\right)}\log(1-{\hat{p}}_{kg}^{*})\\ \;+\sum_{g=1}^{G}I_{\left(X_{g}^{*}>0\right)}X_{g}^{*}\log(d_{kg})+\log\pi_{k}+C. \end{array}$$

### 2.5. Zero-Inflated Negative Binomial Logistic Discriminant Analysis

#### 2.5.1. Model

In this section, we extend the zero-inflated Poisson distribution to the zero-inflated negative binomial distribution and propose ZINBLDA to model overdispersion data with excess zeros. The distribution is expressed as

$$X_{ki_{k}g}\sim \;\left\{ \begin{array}{l} \delta_{\left\{0\right\}},\;p_{ki_{k}g},\\ \text{NB}(\mu_{ki_{k}g},\phi_{g}^{\prime }),\;(1-p_{ki_{k}g}). \end{array}\right.$$

Thus, the probability of *X*_*ki*_*k*_*g*_ is written as

$$P(X_{ki_{k}g})=\left\{ \begin{array}{l} p_{ki_{k}g}+(1-p_{ki_{k}g})(\frac{1}{1+\mu_{ki_{k}g}{\phi^{\prime }}_{g}})\phi_{g}^{\prime }{}^{-1},\;X_{ki_{k}g}=0,\\ (1-p_{ki_{k}g})\frac{\Gamma (X_{ki_{k}g}+\phi_{g}^{\prime }{}^{-1})}{X_{ki_{k}g}!\Gamma (\phi_{g}^{\prime }{}^{-1})}\;(\frac{\mu_{ki_{k}g}{\phi^{\prime }}_{g}}{1+\mu_{ki_{k}g}{\phi^{\prime }}_{g}}{)}^{X_{ki_{k}g}}(\frac{1}{1+\mu_{ki_{k}g}{\phi^{\prime }}_{g}})\phi_{g}^{\prime }{}^{-1},\;X_{ki_{k}g}>0. \end{array}\right.$$

The probability density function of *X*_*ki*_*k*_*g*_ = *x*_*ki*_*k*_*g*_ is

(7) $$\begin{array}{l} P(X_{ki_{k}g}=x_{ki_{k}g})=\text{​}[(1-p_{ki_{k}g})\frac{\Gamma (x_{ki_{k}g}+\phi_{g}^{\prime }{}^{-1})}{x_{ki_{k}g}!\Gamma (\phi_{g}^{\prime }{}^{-1})}(\frac{\mu_{ki_{k}g}{\phi^{\prime }}_{g}}{1+\mu_{ki_{k}g}{\phi^{\prime }}_{g}}{)}^{x_{ki_{k}g}}\\ \;{\left(\frac{1}{1+\mu_{ki_{k}g}{\phi^{\prime }}_{g}}\right)}^{\phi_{g}^{\prime }{}^{-1}}{]}^{I_{\left(x_{ki_{k}g}>0\right)}} \end{array}$$

(8) $${\left[p_{ki_{k}g}+(1-p_{ki_{k}g}){\left(\frac{1}{1+\mu_{ki_{k}g}{\phi^{\prime }}_{g}}\right)}^{\phi_{g}^{\prime }{}^{-1}}\right]}^{I_{\left(x_{{}_{ki_{k}g}}=0\right)}}$$

By Bayes' rule, we obtain the discriminant score $d_{k}\left(x^{*}\right)$ of ZINBLDA using

(9) $$\begin{array}{l} d_{k}(x^{*})=\sum_{g=1}^{G}I_{\left(X_{g}^{*}=0\right)}\log[(1-{\hat{p}}_{kg}^{*})(\frac{1}{1+s^{*}\lambda_{g}d_{kg}{\phi^{\prime }}_{g}}{)}^{\phi_{g}^{\prime }{}^{-1}}\\ \;+{\hat{p}}_{kg}^{*}]+\sum_{g=1}^{G}I_{\left(X_{g}^{*}>0\right)}\log[(1-{\hat{p}}_{kg}^{*})\\ \;+\sum_{g=1}^{G}I_{\left(X_{g}^{*}>0\right)}X_{g}^{*}[\log d_{kg}-\log(1+s^{*}\lambda_{g}d_{kg}\phi_{g}^{\prime })]\\ \;-\sum_{g=1}^{G}I_{\left(X_{g}^{*}>0\right)}\phi_{g}^{\prime }{}^{-1}\log(1+s^{*}\lambda_{g}d_{kg}\phi_{g}^{\prime })\\ \;+\log\pi_{k}+C. \end{array}$$

#### 2.5.2. Parameters Estimation

Next, we estimate the parameters in the ZINBLDA model, which includes the class difference parameter *d*_*kg*_, size factors *s*_*i*_*k*__ and s^*^, dispersion parameter ${\phi \prime }_{g},$ and the probability of excess zeros *p*_*ki*_*k*_*g*_.

##### 2.5.2.1. Class Difference Parameter Estimation

Similar to the former three methods, to estimate *d*_*kg*_ we first obtain the maximum likelihood estimation ${\hat{d}}_{kg}=\left(\overset{n_{k}}{\underset{i_{k}=1}{\sum }}X_{i_{k}g}\right)/\left(\overset{n_{k}}{\underset{i_{k}=1}{\sum }}s_{i_{k}}\lambda_{g}\right)$ and then take a Gamma(β, β) prior in case of $\overset{n_{k}}{\underset{i_{k}=1}{\sum }}X_{i_{k}g}=0$. Therefore, the posterior mean

$${\hat{d}}_{kg}=\left(\overset{n_{k}}{\underset{i_{k}=1}{\sum }}X_{i_{k}g}+\beta \right)/\left(\overset{n_{k}}{\underset{i_{k}=1}{\sum }}s_{i_{k}}\lambda_{g}+\beta \right)$$

is our estimation. For convenience and due to the small influence of β on the estimation result, we assume β = 1 in this work.

##### 2.5.2.2. Size Factor Estimation

The total number of reads between samples differs due to various sequencing depths. Generally, data must be normalized by their size factor. The three existing classification methods (PLDA, NBLDA, and ZIPLDA) use three different normalization methods: total count (Dillies et al., 2013), median ratio (Love et al., 2014), and quantile (Bullard et al., 2010). Note that there is little difference in the performance of classification among the three normalization methods. In this work, we use total count to estimate the size factor for convenience. Therefore, the estimation of size factor ŝ_*i*_*k*__ for the training data is

$${\hat{s}}_{i_{k}}=\frac{\sum_{g=1}^{G}X_{i_{k}g}}{\sum_{k=1}^{K}\sum_{i_{k}=1}^{n_{k}}\sum_{g=1}^{G}X_{i_{k}g}},$$

and the estimation of size factor ŝ^*^ for the testing data is

$${\hat{s}}^{*}=\frac{\sum_{g=1}^{G}X_{g}^{*}}{\sum_{k=1}^{K}\sum_{i_{k}=1}^{n_{k}}\sum_{g=1}^{G}X_{i_{k}g}}.$$

##### 2.5.2.3. Dispersion Parameter Estimation

Since ZINBLDA assumes that data follow a mixture distribution rather than a negative binomial distribution, the method used to estimate the dispersion parameter in NBLDA is not applicable in this case. Therefore, we used the maximum likelihood to estimate ${\phi \prime }_{g}$. Based on equation (8), the log likelihood function of ZINBLDA is

(10) $$\begin{array}{l} L=\sum_{g=1}^{G}\{I_{(}{{}_{x}}_{{}_{ki_{k}g}=0)}\log[{\hat{p}}_{ki_{k}g}+(1-{\hat{p}}_{ki_{k}g}))(\frac{1}{1+{\hat{\mu }}_{ki_{k}g}{\phi^{\prime }}_{g}}{)}^{\phi_{g}^{\prime -1}}]\\ \;+I_{\left(x_{ki_{k}g}>0\right)}[\log(1-{\hat{p}}_{ki_{k}g})+\log\Gamma (x_{ki_{k}g}+\phi_{g}^{\prime }{}^{-1})\\ \;-\log\Gamma (\phi_{g}^{\prime }{}^{-1})-\log\Gamma (x_{ki_{k}g}!)\\ \;+x_{ki_{k}g}\log{\hat{\mu }}_{ki_{k}g}{\phi^{\prime }}_{g}-x_{ki_{k}g}\log(1+{\hat{\mu }}_{ki_{k}g}{\phi^{\prime }}_{g})\\ \;+\phi_{g}^{\prime }{}^{-1}\log(1+{\hat{\mu }}_{ki_{k}g}\phi_{g}^{\prime })]\}. \end{array}$$

Because the parameter *p*_*ki*_*k*_*g*_ must also be estimated, we cannot directly take the partial derivatives and let the result equal zero to get the estimation of dispersion parameter $\hat{\phi }\prime_{g}$ in formula (10). Therefore, we first set an initial value for parameters *p*_*ki*_*k*_*g*_ and ϕ_*g*_, and then we used the PORT routines optimization method (David, 1990) to get the estimation value $\hat{\phi }\prime_{g}$.

##### 2.5.2.4. The Probability of Excess Zeros Estimation

Assuming the data for the classifier follow a zero-inflated mixture distribution, we needed to estimate the probability of excess zeros in the distribution. Based on the process proposed by Zhou et al. (2018), we assumed that the probability of zeros, the mean of the genes, and the sequencing depth have the following logistic relation:

(11) $$\begin{array}{l} log\left\{\frac{P\left(X_{ki_{k}g}=0\right)}{1-P\left(X_{ki_{k}g}=0\right)}\right\}=\alpha +\beta_{1}\left(\frac{N_{ki_{k}}}{N_{1i_{1}}}\right)+\beta_{2}\mu_{ki_{k}g}. \end{array}$$

Replacing *P*(*X*_*ki*_*k*_*g*_ = 0) in model (11) with $p_{ki_{k}g}+\left(1-p_{ki_{k}g}\right){\left(\frac{1}{1+\mu_{ki_{k}g}\phi_{g}^{{}^{\prime }}}\right)}^{\phi_{g}^{{}^{\prime }-1}}$, we get

$${\hat{p}}_{ki_{k}g}=\frac{\;p_{1}-(1+p_{1}){\left(\frac{1}{1+\mu_{ki_{k}g}{\hat{\phi }}_{g}^{\prime }}\right)}^{{\hat{\phi }}_{g}^{\prime -1}}}{\left(1+p_{1})[1-{\left(\frac{1}{1+\mu_{ki_{k}g}{\hat{\phi }}_{g}^{\prime }}\right)}^{{\hat{\phi }}_{g}^{\prime -1}}\right]},$$

where $p_{1}=exp\left\{\alpha +\beta_{1}\left(\frac{N_{ki_{k}}}{N_{1i_{1}}}\right)+\beta_{2}\mu_{ki_{k}g}\right\}$; $N_{ki_{k}}=\overset{G}{\underset{g=1}{\sum }}X_{ki_{k}g}$; and α, β_1_, and β_2_ are coefficients in the logistic model (11).

### 2.6. Transformation Relation

Note that the above four models can transform into one another under some conditions.

(1) From the discriminant score function of NBLDA (formula 5), we found that if $s^{*}\lambda_{g}d_{kg}$ is bounded and ϕ_*g*_ → 0, then $\log\left(1+s^{*}\lambda_{g}d_{kg}\phi_{g}\right)\to 0$ and $\phi_{g}^{-1}\log\left(1+s^{*}\lambda_{g}d_{kg}\right)\to s^{*}\lambda_{g}d_{kg}$. Therefore, the discriminant score of NBLDA approaches that of PLDA (formula 3). That is, the NBLDA classifier reduces to the PLDA classifier when the dispersion value tends to zero.

(2) For the discriminant score function of ZIPLDA (formula 6), when ${\hat{p}}_{kg}^{*}\to 0$, then $\log({\hat{p}}_{kg}^{*}+(1-{\hat{p}}_{kg}^{*})e^{-d_{kg}s^{*}\lambda_{g}})\to -d_{kg}s^{*}\lambda_{g}$, and the discriminant score of ZIPLDA approaches that of PLDA. Thus, with the probability of zeros decreased to zero, the ZIPLDA score reduces to the PLDA score.

(3) Similarly, for the discriminant score of ZINBLDA (formula 9), when ${\phi \prime }_{g}\to \;0,$ then $\phi_{g}^{\prime -1}\log(1+s^{*}\lambda_{g}d_{kg}\phi \prime_{g})\to d_{kg}s^{*}\lambda_{g}$ and $(1+s^{*}\lambda_{g}d_{kg}\phi \prime_{g}{)}^{-\phi_{g}^{\prime -1}}\to \;exp\{-d_{kg}s^{*}\lambda_{g}\}.$ That is, when dispersion tends to zero, the discriminant score of ZINBLDA reduces to that of the ZIPLDA. Furthermore, if ${\hat{p}}_{kg}^{*}\to 0$, then $\log[(1-{\hat{p}}_{kg}^{*})(\frac{1}{1+s^{*}\lambda_{g}d_{kg}{\phi^{\prime }}_{g}}{)}^{\phi_{g}^{\prime -1}}+{\hat{p}}_{kg}^{*}]\to -\phi_{g}^{\prime -1}\log(1+s^{*}\lambda_{g}d_{kg}{\phi \prime }_{g})$. Therefore, when the probability of δ_{0}_ tends to zero, the ZINBLDA classifier reduces to the NBLDA classifier.

**Figure 1:**
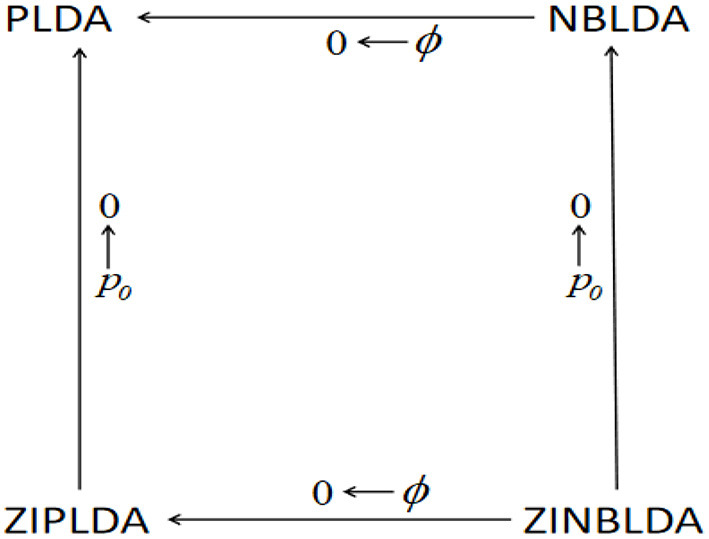
The transformation relation between the four methods. Given a sufficient sample size, the four models can transform into one another according to the value of dispersion parameter ϕ and the average probability of excess zeros *p*_0_.

Figure 1 shows the above transformation relations, where ϕ denotes the dispersion parameter, and *p*_0_ denotes the average probability of excess zeros. Starting at the bottom right of the figure and going clockwise, ZINBLDA reduces to ZIPLDA as ϕ → 0, and ZIPLDA reduces to PLDA as *p*_0_ → 0. Likewise, starting at the bottom right corner and going counterclockwise, ZINBLDA reduces to NBLDA as *p*_0_ → 0, and NBLDA reduces to PLDA as ϕ → 0. The transformation relationship between the four classification methods indicates that for data without dispersed biological replicates and excess zeros, PLDA may perform better than the other methods. However, NBLDA is good at dealing with overdispersion data, while ZIPLDA is designed to handle data with excess zeros. For data with excess zeros and dispersed biological replicates, ZINBLDA may be the optimal choice.

## 3. Simulation Studies

We evaluated the performance of the four methods by conducting simulations in various scenarios. We also built a decision tree to help us select the optimal classifier from the four methods for a new dataset.

### 3.1. Simulation Design

To ensure a fair comparison between the four classifiers, we followed the same process as Zhou et al. (2018) and generated simulation data from the following negative binomial distribution:

$$\begin{array}{l} X_{ki_{k}g}\;\sim \text{ NB}\left(d_{kg}s_{i_{k}}\lambda_{g},\phi \right). \end{array}$$

We set *K* = 2 to illustrate the binary classification, and each class included about *n*/2 samples. We also considered multiple classifications with *K* = 3, with each class including approximately *n*/3 samples. The rest of the distribution parameters were as follows: the size factors *s*_*i*_*k*__ had a uniform distribution at [0.2, 2.2], the λ_*g*_ values had an exponential distribution with an expectation of 25, and the log*d*_*kg*_ values had a normal distribution with a location of 0 and scale of σ^2^ (where σ = 0.2). In the simulation studies, the *DE rate* represented the proportion of differentially expressed genes, and *p* was the number of genes in the samples. For simplicity, we denoted *p*_0_ as the probability of excess zeros. In each simulation study, we changed one parameter and fixed the others, then compared the misclassification rates of the four classifiers. We specified the values for *p*, *DE rate*, *p*_0_, ϕ, and *n* in each simulation study. Each simulation was repeated 1,000 times, and the average misclassification rates were calculated for the four methods.

### 3.2. Simulation Results

**Figure 2:**
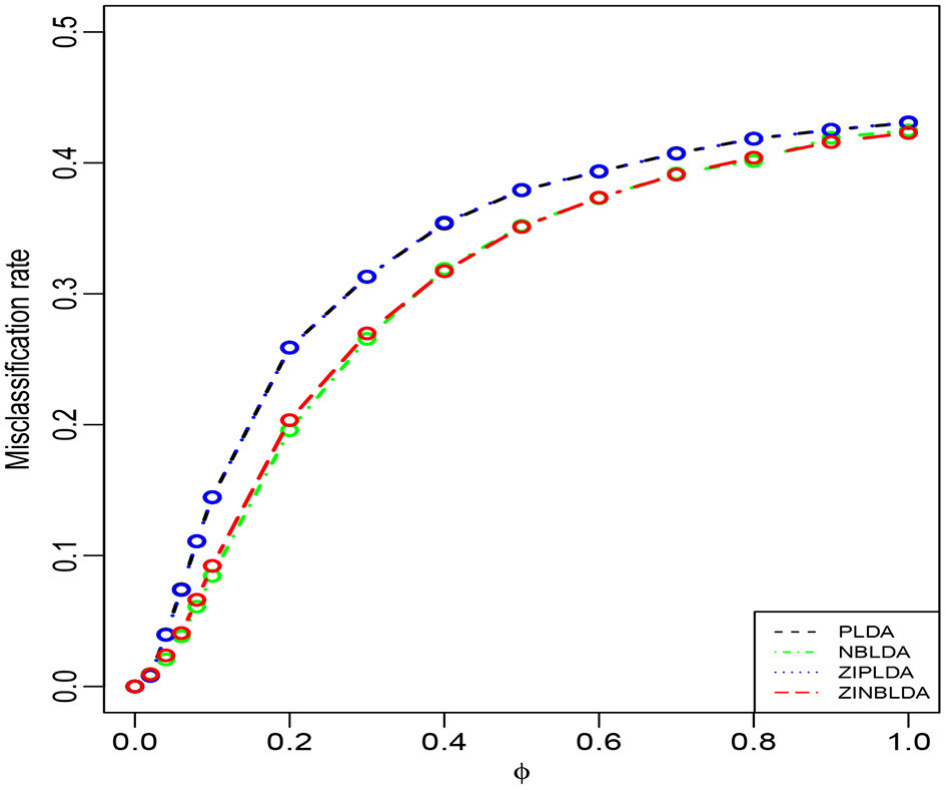
The misclassification rates of the four methods with different dispersions (Study 1). Here, σ = 0.2, *p* = 100, *n* = 50, *p*_0_ = 0, and *DE rate* = 0.2.

Study 1 investigated the impact of the dispersion parameter on the performance of the four classification methods. Considering a binary classification, we set the probability of excess zeros of data to 0 and generated 50 training and 50 testing samples. Each sample included 100 genes, 20% of which were DE genes. Figure 2 shows the average misclassification rates of the four methods with different dispersions. Overall, the misclassification rates of the four classifiers decreased when the dispersion parameters changed from 1 to 0. PLDA and ZIPLDA showed similar performance, and both were slightly worse than NBLDA and ZINBLDA in different dispersion settings. However, when the dispersion was reduced to zero, the misclassification rates of all four methods tended to zero. From the expressions of the negative binomial and Poisson distributions, the former reduced to the latter when the dispersion parameter was reduced to zero, which indicates that NBLDA and ZINBLDA (which are based on negative binomial distribution) are more suitable for classifying overdispersion data. In addition, we changed the probability of excess zeros of simulation data from 0 to 0.1, and the other parameters remained the same. Supplementary Figure 1 shows that when dispersion changed the value from 0 to 1, ZINBLDA outperformed the other methods. However, NBLDA and PLDA performed worse than ZINBLDA and ZIPLDA when the dispersion tended to zero. This result indicates that the probability of excess zeros has a major effect on the performance of the four methods.

**Figure 3:**
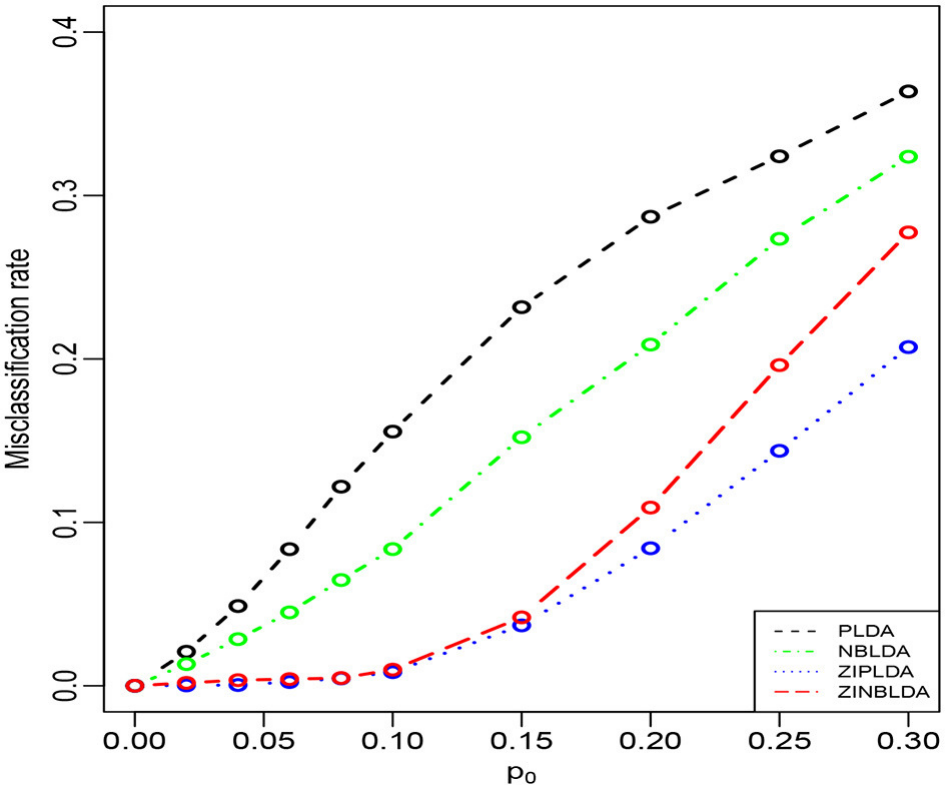
The misclassification rates of the four methods with different probabilities of excess zeros (Study 2). Here, σ = 0.2, *p* = 100, *n* = 50, ϕ = 0, and *DE rate* = 0.2.

Study 2 investigated the performance of the four methods with different probabilities of excess zeros. In this study, we set the dispersion parameter to 0, and *p*_0_ ∈ [0.1, 0.3]; the rest of the parameters were the same as in Study 1. Figure 3 shows that the average misclassification rates of the four classifiers increased as the probability of excess zeros increased. ZIPLDA performed slightly better than ZINBLDA when *p*_0_ tended to 0. The performance of these two classifiers was far better than the other two classifiers, and PLDA performed the worst with different probabilities of excess zeros. This result demonstrates that ZIPLDA and ZINBLDA (which are designed for excess zeros) have a clear advantage over the other two methods when classifying data with excess zeros. Setting ϕ = 0 could explain why ZIPLDA performed slightly better than ZINBLDA. In addition, when we reduced the sample size from 50 to 8, the result (Supplementary Figure 2) showed that ZIPLDA still performed the best out of the four classifiers; however, ZINBLDA performed the worst in this case, which indicates that the sample size has a major effect on the performance of ZINBLDA.

**Figure 4:**
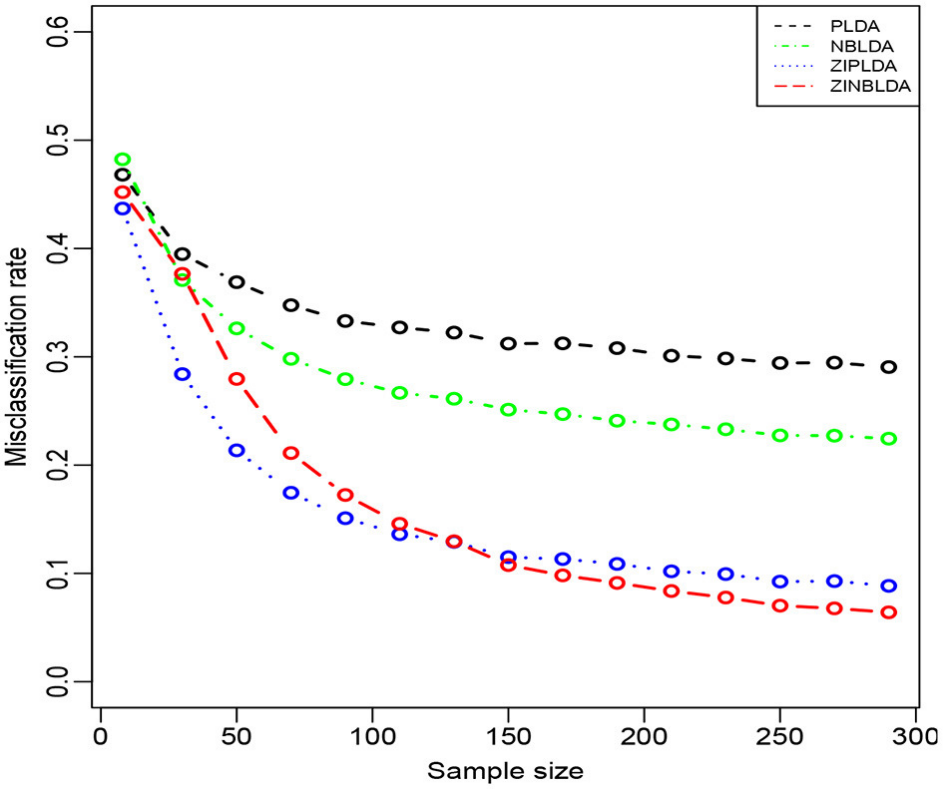
The misclassification rates of the four methods with different sample sizes (Study 3). Here, σ = 0.2, *p* = 100, ϕ = 0, *p*_0_ = 0.3, and *DE rate* = 0.2.

Figure 4 shows the performance of the four classification methods when the sample size changes. In Study 3, we set the probability of excess zeros to 0.3, and the sample size was gradually changed from 8 to 300. The rest of the parameters were the same as in Study 2. The overall misclassification rates gradually declined to nearly a constant value for all four classifiers when the sample size increased. ZIPLDA showed superiority over the other methods when the sample size was less than 130, and ZINBLDA attained a lower misclassification rate when the sample size was over 150. The same pattern existed between NBLDA and PLDA. When the sample size was less than 20, PLDA had a lower misclassification rate; however, NBLDA yielded a lower value when the sample size increased. The results illustrate that sample size has a huge impact on the performance of ZINBLDA and NBLDA, and ZINBLDA outperformed the other methods when a sufficient sample size was available. The reason for this may be that ZINBLDA requires a minimal number of samples to estimate the parameters in the model.

**Figure 5:**
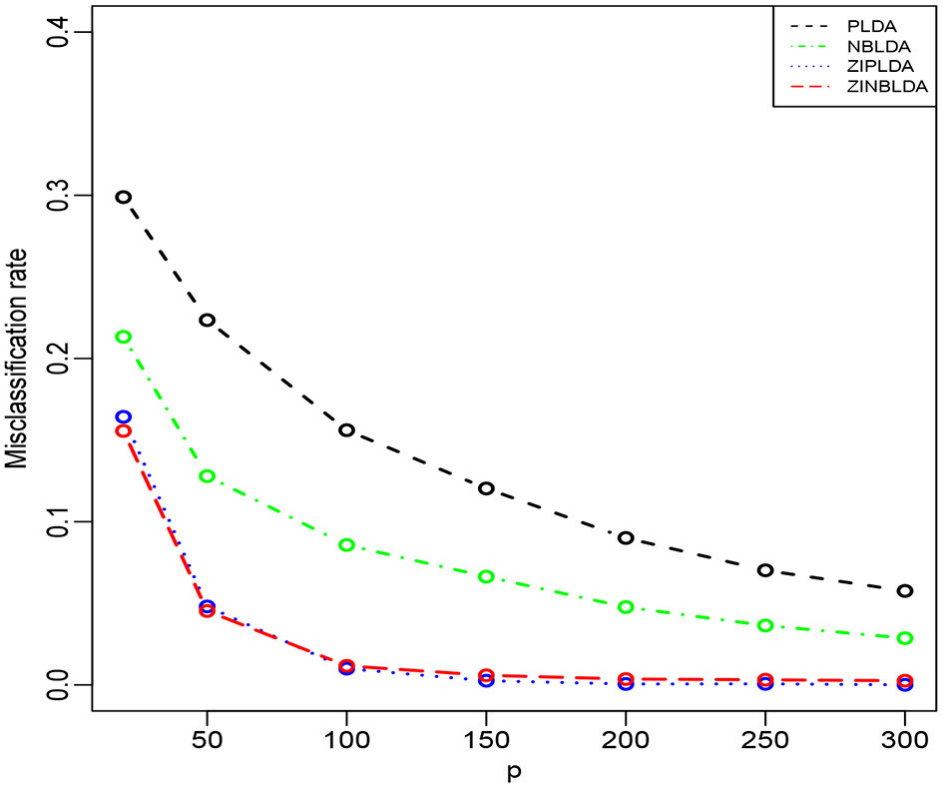
The misclassification rates of the four methods with different numbers of genes (Study 4). Here, σ = 0.2, *n* = 50, ϕ = 0, *p*_0_ = 0.1, and *DE rate* = 0.2.

In the above three studies, we fixed the gene number at 100. In Study 4, we changed the number of selected genes and evaluated the performance of the four classifiers. The parameters were the same as in the former studies except ϕ = 0 and *p*_0_ = 0.1. Figure 5 shows that the misclassification rates of the four methods declined as the number of genes selected increased. ZINBLDA and ZIPLDA showed similar performance and outperformed the other two methods, and PLDA performed the worst of the four methods. In Supplementary Figure 3, we changed the data dispersion from 0 to 0.2. A lower misclassification rate was obtained by ZINBLDA and NBLDA, and PLDA again performed the worst of the four methods. This result agrees with the conclusion that dispersion affects the performance of PLDA and ZIPLDA.

**Figure 6:**
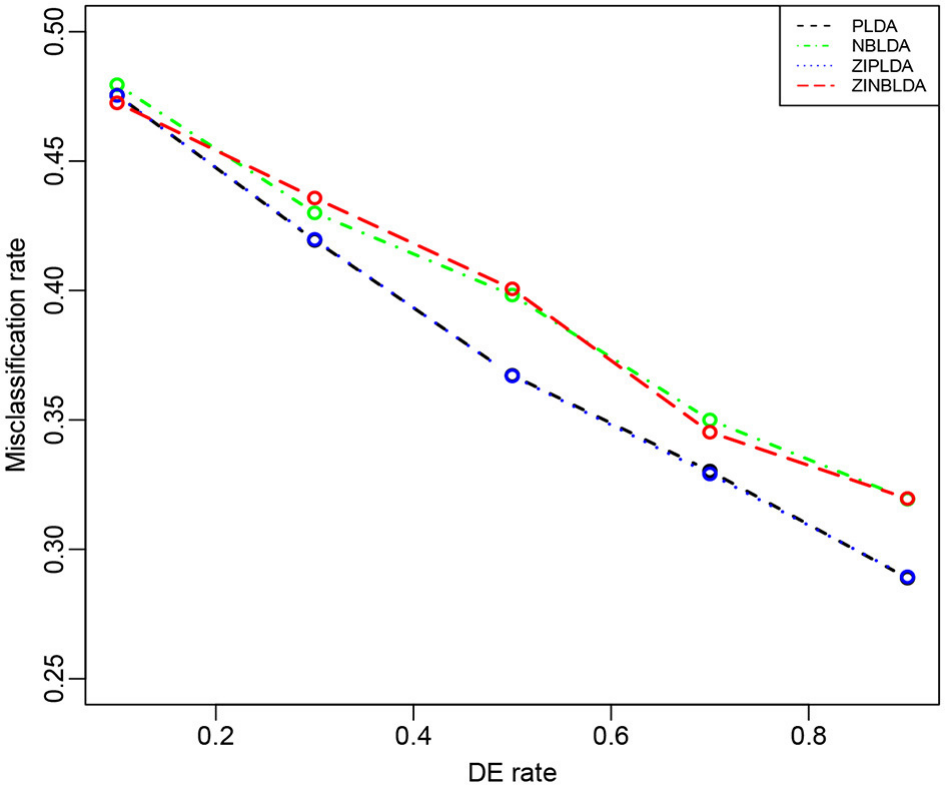
The misclassification rates of the four methods with different probabilities of differential expression genes (Study 5). Here, σ = 0.2, *p* = 100, *n* = 8, ϕ = 0.5, and *p*_0_ = 0.

Study 5 investigated the influence of the probability of differential expression in the selected genes on the performance of the four classifiers. In this study, we set the dispersion parameter to 0.5, the probability of excess zeros was set at 0, and 100 genes were selected for all eight samples. Figure 6 shows that the overall misclassification rates of the four methods decreased as the DE rate increased. PLDA and ZIPLDA showed similar performances, and both performed better than NBLDA and ZINBLDA with different DE rates. ZINBLDA and NBLDA performed nearly same with different probabilities of DE genes. This result demonstrates that the sample size has a marked impact on the performance of the four classifiers. In addition, Supplementary Figure 4 shows that when the dispersion was set to 0.2 and the probability of zeros to 0.1, ZIPLDA performed remarkably better than the other methods with an increasing number of DE rates, followed by PLDA and then NBLDA, ZINBLDA performed the worst. This indicates that excess zeros in the data enable ZIPLDA to perform better than PLDA, and the sample size affects the performance of ZIPLDA notably.

We also conducted Simulation Studies 1–5 using multiple classifications (*K* = 3). Supplementary Figures 5–9 show the performance of the four methods. The parameters were the same as in Studies 1–5 except for the sample sizes. We set *n* = 75 in Supplementary Figures 5, 6, 8; *n* = 12 in Supplementary Figure 9; and *n* = 450 in Supplementary Figure 7 and compared the results with those of Studies 1–5. The performance of the four classifiers remained the same as in the binary classification.

In the simulation studies conducted above, the performance of the four classifiers was related to the attributes of the dataset, including sample size *n*, dispersion parameter ϕ, and the probability of excess zeros *p*_0_. In the final simulation study, we considered a binary classification with three changeable parameters and compared the performance of the four methods for different combinations of those three parameters. We still selected 100 genes, 40% of which were DE genes. The probability of excess zeros was set at 0.001, 0.1, or 0.3, and the sample size was 8, 50, or 100. The dispersion parameters changed from 0.001 to 0.1 to 1 with every 0.2 steps. The average misclassification rates of the four methods are shown in Supplementary Figure 10. To clarify display the result, Supplementary Table 1 shows the concrete values of each misclassification rate. Comparing the results of the three panels in each column, we found that for the first column (sample size of 8), the overall misclassification rates of the four methods increased when the probability of excess zeros increased from 0.001 to 0.3. When the probability of excess zeros was equal to 0.3, the misclassification rates approached 50%. When *p*_0_ = 0.001, the performance of ZIPLDA and PLDA was better than NBLDA and ZINBLDA. However, when *p*_0_ = 0.1, ZIPLDA outperformed the other methods, which indicates that ZIPLDA is more suitable for handling data with a small probability of excess zeros, and the sample size has less of an impact on it. When the sample size was increased to 50 (the second column), the overall performance of ZINBLDA was slightly better than that of ZIPLDA except when ϕ was small and *p*_0_ = 0.3. The reason for this may be that there were not enough samples to estimate the parameters of ZINBLDA. Therefore, when the sample size increased to 100 (the third column), that ZINBLDA yielded a lower or equal misclassification rate compared to the other methods, which indicates that ZINBLDA can achieve the best classification result as long as enough samples are available. The performance of ZIPLDA also improved when *p*_0_ increased from 0.1 to 0.3 due to the increase in the probability of excess zeros.

### 3.3. Optimal Classifier Selection

To select an optimal classification method for different datasets, we built a decision tree and a random forest. A decision tree is a machine learning algorithm that is widely used in many scenarios because of its accuracy for the current algorithms. As its name implies, a decision tree is a decision support tool that uses a tree-like model. It is comprised of nodes and branches, and each sample is tested on an internal node. The outcome of the test determines which branch is followed, and this procedure continues until the leaf node that holds the class label of this sample is reached. Random forest is an ensemble of a decision tree, and it can achieve a more stable result than a decision tree.

**Figure 7:**
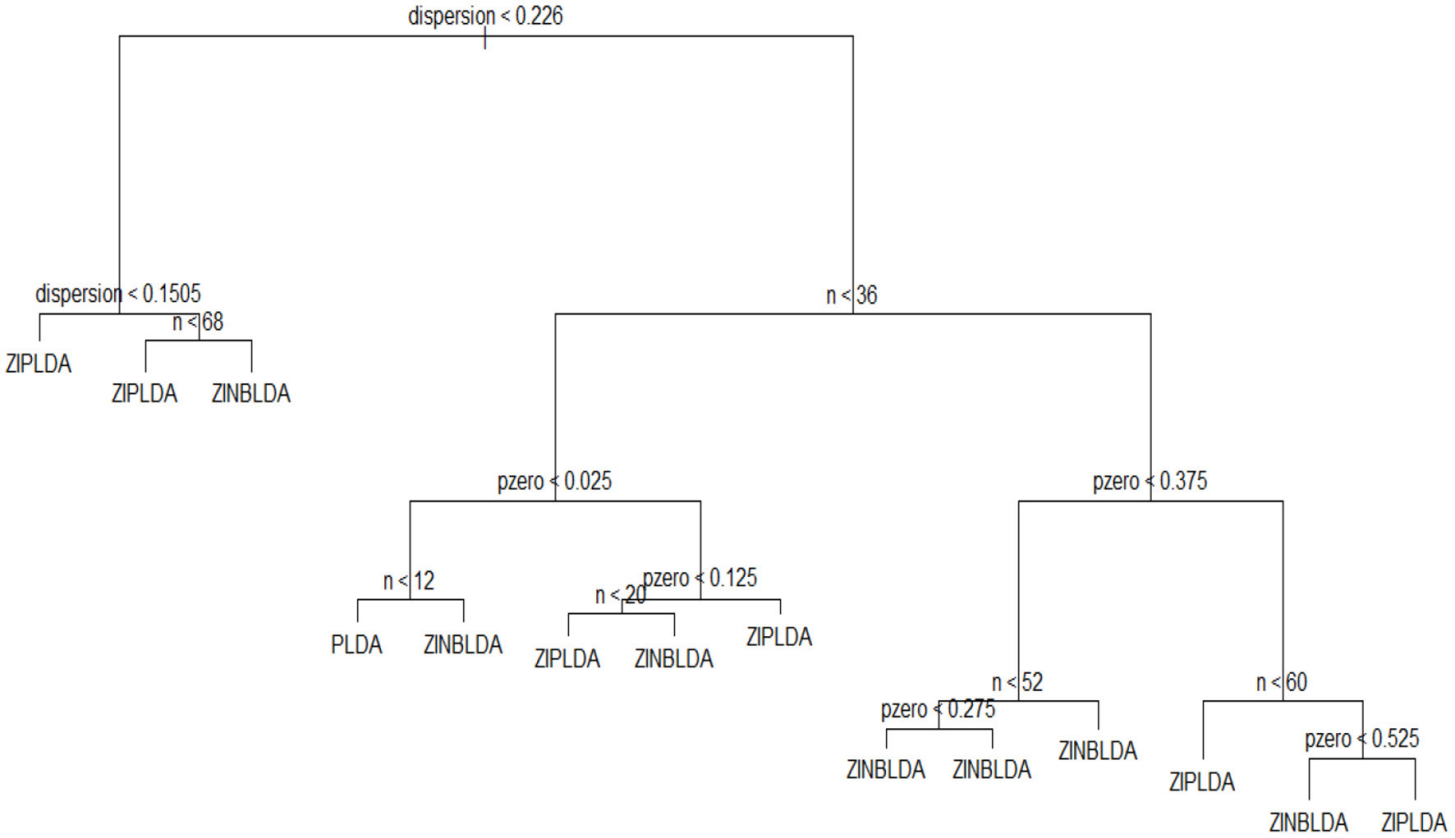
The decision tree model used to choose an optimal classifier.

To employ a tree-like model to select the optimal classifier, the chosen features were the sample number *n*, dispersion ϕ, and probability of excess zeros *p*_0_, and *y* was regarded as the optimal classification method. The parameter region was divided to assign the value of the feature vector. The values of sample size *n* ranged from 8 to 100 with a step size of 8. The dispersion parameter ranged from 0.001 to 1.001 at intervals of 0.1. The probability of excess zeros *p*_0_ ranged from 0 to 0.6 with a step size of 0.05. For each calculation, we took one value from each parameter set to generate the simulation data, allowing for multiple combinations of these three parameters. This procedure was repeated 1,000 times, and the classifier corresponding to the smallest value of the average misclassification rate was regarded as the optimal classification method. We used the obtained data to train a decision tree, and Figure 7 displays the classification result. This model fits the data very well, with a misclassification rate of only 7.4%. To use this model, we only need to know or estimate the values of the three parameters, then use the conditional control statements in the decision tree to distinguish in each internal node, which will result in the optimal method when the leaf node is reached. In this way, this model can be used to help choose the optimal classification method. Similarly, we can obtain a random forest with a lower misclassification rate (2.2%). The classification results of decision tree and random forest are saved in R scripts, which could be used to choose the optimal classifier when inputting the parameters of dataset.

## 4. Application to Real data

We further compared the four methods by analyzing two real datasets: GSE86507 and TCGA-LIHC (Liver Hepatocellular Carcinoma). The details of these two RNA-seq datasets are as follows.

Woo et al. (2017) created the GSE86507 dataset to compare gene expression between two mouse models, Pkd1f/f: HoxB7-cre mice and Pkd2f/f: HoxB7-cre mice. Each group includes 18 samples, and there are a total of 29,996 transcripts in this dataset. It contains about 17.74% zeros of all numerical values.

The dataset TCGA-LIHC contains two groups of samples: the normal group (340 samples) and the cancerous group (50 samples). There are 60,487 genes in this dataset, which contains about 43.24% zeros of all numerical values.

We chose to classify parts of genes since the majority of genes in a dataset are not differentially expressed and thus do not contribute to the sample classification. Including entire genes in the model would reduce the classification accuracy and increase the computational complexity. Thus, selecting parts of genes not only improves the accuracy of classification but saves computation time. Following the steps outlined by Dudoit et al. (2002), we selected genes by first calculating the ratio of the sum of the squares between groups and within groups for each gene, then sorted all of the genes according to the ratio from greatest to least, and finally selected a certain number of genes for downstream analysis.

**Figure 8:**
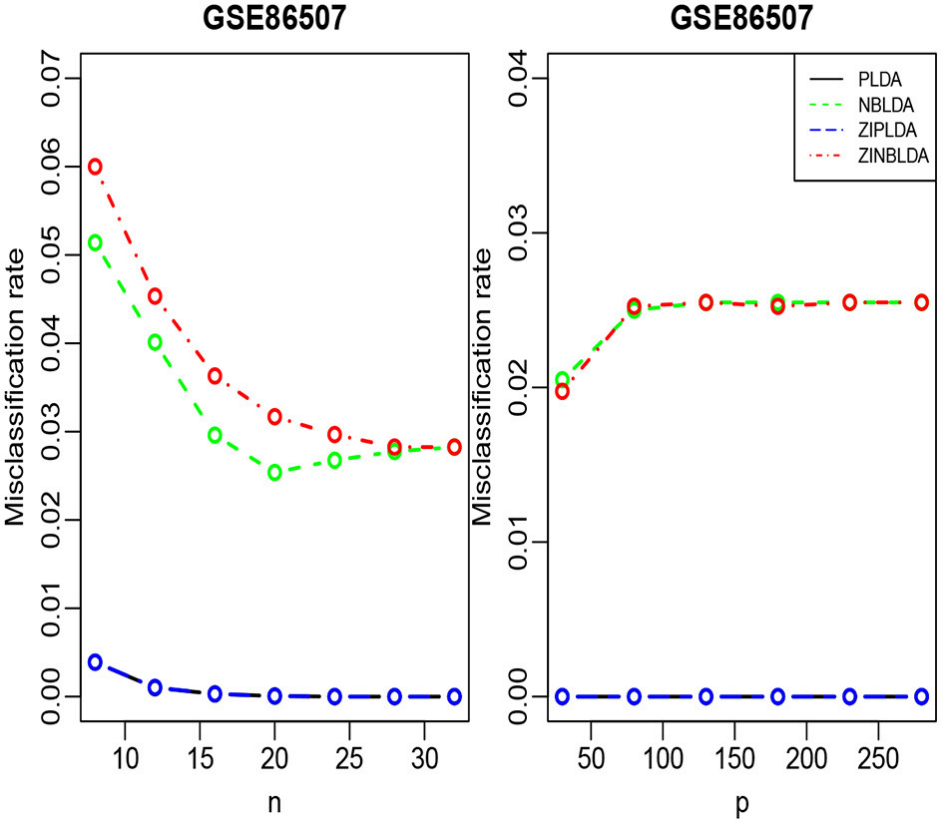
The misclassification rates of the four classifiers for the GSE86507 dataset.

**Figure 9:**
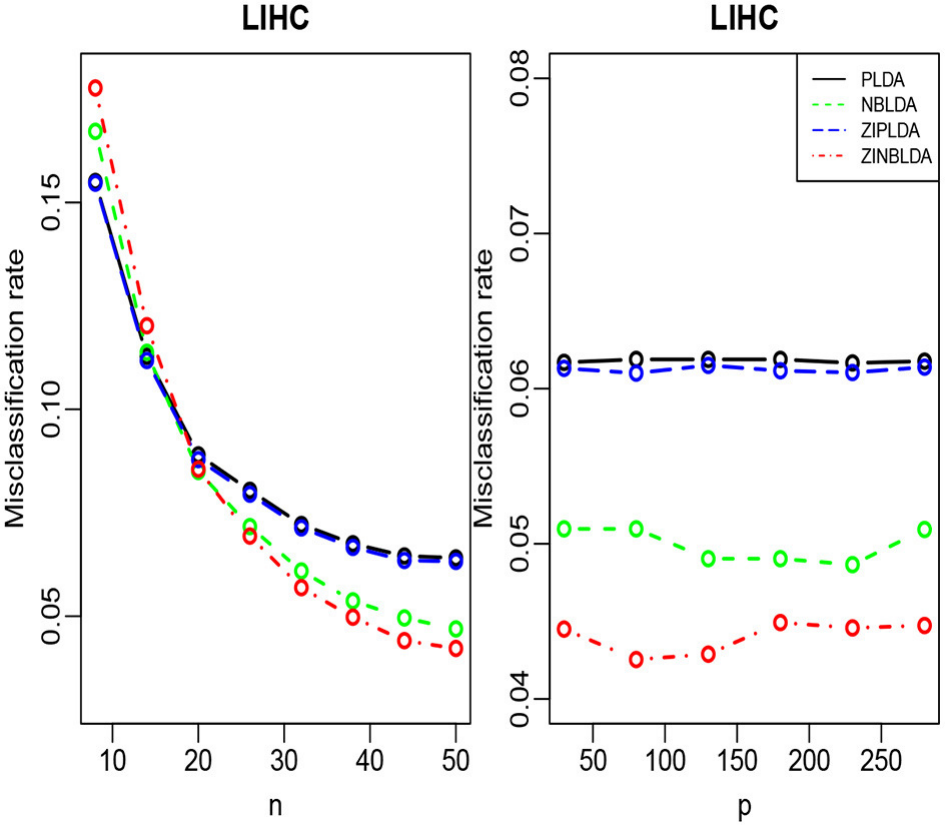
The misclassification rates of the four classifiers for the LIHC dataset.

We randomly split the data into a training set and test set, with both datasets containing all classes. We selected the 300 most differentially expressed genes to train the model. This procedure was repeated 1,000 times, and the average misclassification rates for each method were recorded. The left panels of Figures 8, 9 show that for the test data, the average misclassification rates of the four methods decreased as the number of training data gradually increased. For the GSE86507 dataset, the misclassification rates of PLDA and ZIPLDA were lower than NBLDA and ZINBLDA, both of which were close to zero. However, for the TCGA-LIHC dataset, PLDA and ZIPLDA were superior to NBLDA and ZINBLDA when the sample size was small. As the training sample size increased, the misclassification rates of NBLDA and ZINBLDA decreased remarkably, and ZINBLDA outperformed the other three methods for a large sample size. We also evaluated the classification performance of the four methods by fixing 30 training sets and gradually increasing the number of selected DE genes. The right panel of Figure 8 shows that PLDA and ZIPLDA outperformed the other two methods, whereas the right panel of Figure 9 shows the superiority of ZINBLDA over the other methods in this case.

To assess the efficiency of the decision tree model, we estimated the dispersion and probability of excess zeros for the two datasets. The estimated dispersion of GSE86507 was ϕ = 0.12, and the probability of excess zeros was 0.5%, which indicates that the dataset has slight overdispersion and almost no excess zeros. The estimated dispersion of TCGA-LIHC was ϕ = 1.12, and the probability of excess zeros was 8%, which indicates that the dataset has high overdispersion and many excess zeros. According to the conclusions in section 2.6, PLDA should perform better with the GSE86507 dataset, and ZINBLDA should be the optimal method to classify the TCGA-LIHC dataset. We used the estimated parameters to select the optimal method according to the conditional control statements in the decision tree model (Figure 7). Based on the result, we recommend selecting ZIPLDA for the GSE86507 dataset and ZINBLDA for the TCGA-LIHC dataset, which coincides with the real analysis results.

## 5. Discussion

RNA-seq data classification is vital to the diagnosis of diseases. In this work, we extended the existing classification methods and proposed a ZINBLDA method for overdispersion RNA-seq data with an excess of zeros. Concretely, we built a mixture distribution with a point mass at zero and a negative distribution to model the data, and a logistic regression was used to build a relation between the probability of zeros, the mean of the genes, and the sequencing depth. Most importantly, we examined four classification methods from the perspective of their parameters, and we found that these four methods can transform into each other in some cases.

In the simulation studies, we evaluated the performance of the four methods in a wide range of settings. The simulation results showed that different methods perform better for different applications. In addition, we found that the application region of each method is associated with the attributes of the dataset, such as the dispersion, sample size, and probability of excess zeros. Therefore, we built a decision tree to help us select the optimal classification methods in different cases. In the real data analysis, we analyzed two real, next-generation sequencing datasets, and the results further confirmed the theory and simulation conclusions.

Although each of the four methods performed well in certain scenarios, there are numerous issues that remain to be solved, such as single cell RNA-seq data being particularly prone to dropout events due to the relatively shallow sequencing depth per cell. In this case, the existing classification methods may not provide a good result in practice. Therefore, we plan to develop a new classification method that employs deep learning technology to model scRNA-seq data to further improve our current work.

## Data Availability Statement

The dataset GSE86507 for this study can be found in the NCBI repository (https://www.ncbi.nlm.nih.gov/geo/query/acc.cgi?acc=GSE86507), and the dataset TCGA-LIHC for this study can be found in the GDC repository (https://portal.gdc.cancer.gov/projects/TCGA-LIHC).

## Author Contributions

YZ conceived the idea. JZ processed the data and conducted simulation and real dataset experiments. JZ, ZY, and YZ wrote the manuscript. LS, MZ, YZ, and WL revised the manuscript. All authors read and approved the final manuscript.

## Conflict of Interest

The authors declare that the research was conducted in the absence of any commercial or financial relationships that could be construed as a potential conflict of interest.
