# Supplementary material for: Selecting Classification Methods for Small Samples of Next-Generation Sequencing Data
Source: Front Genet. 2021 Mar 4;12:642227. doi: 10.3389/fgene.2021.642227 (PMC7969809; doi:10.3389/fgene.2021.642227)
Supplement: Supplementary file 1 [file Data_Sheet_1.PDF]

Supplementary Material

1 SUPPLEMENTARY DATA

The dataset GSE86507 for this study can be found in the NCBI repository (https://www.ncbi.nlm.nih.gov/geo/query/acc.cgi?acc=GSE86507), and the dataset TCGA-LIHC for this study can be found in the GDC repository (https://portal.gdc.cancer.gov/projects/TCGA-LIHC).

2 SUPPLEMENTARY TABLES AND FIGURES

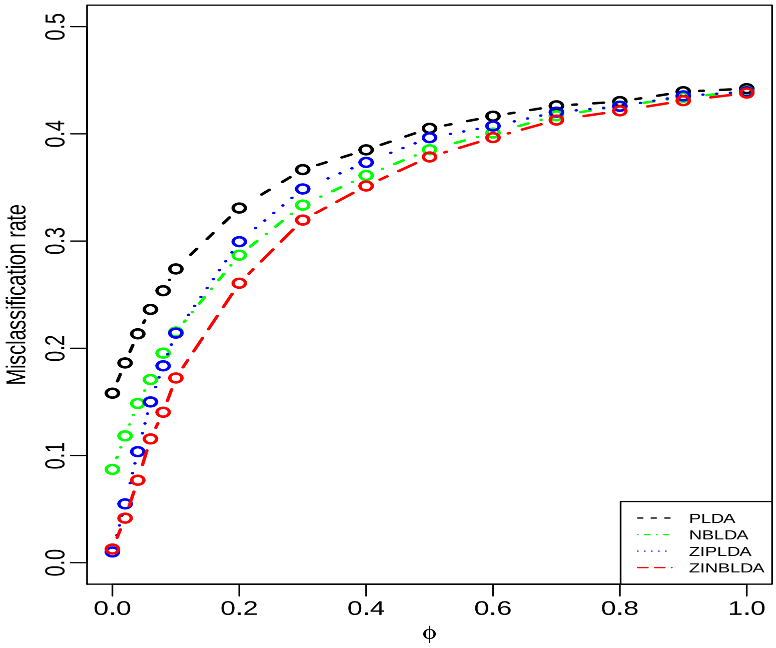

Figure S1. The misclassification rates of the four methods with the different dispersions (Study 1). Here, $K = 2$, $\sigma = 0.2$, $p = 100$, $n = 50$, $p_0 = 0.1$ and $DE\ rate = 0.2$.

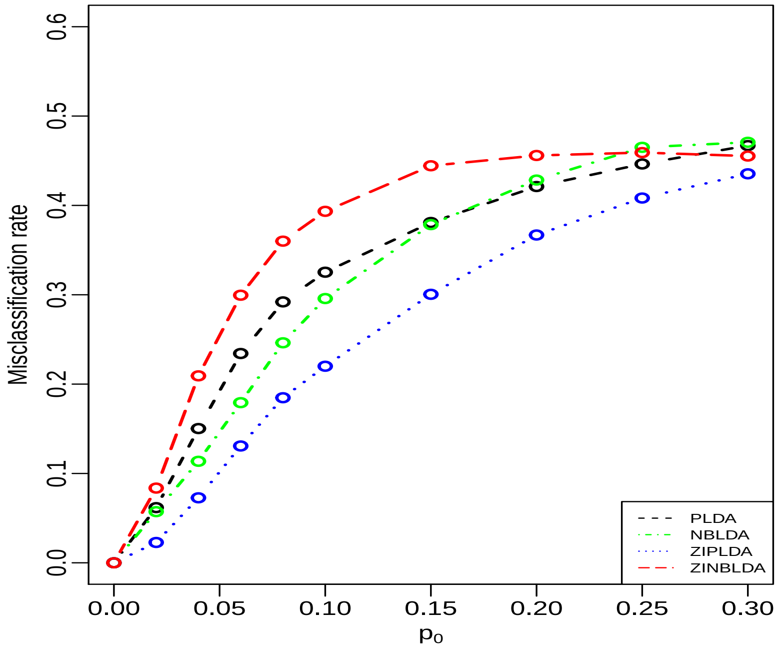

Figure S2. The misclassification rates of the four methods with different probabilities of excess zeros (Study 2). Here, $K = 2$, $\sigma = 0.2$, $p = 100$, $n = 8$, $\phi = 0$ and $DE\ rate = 0.2$.

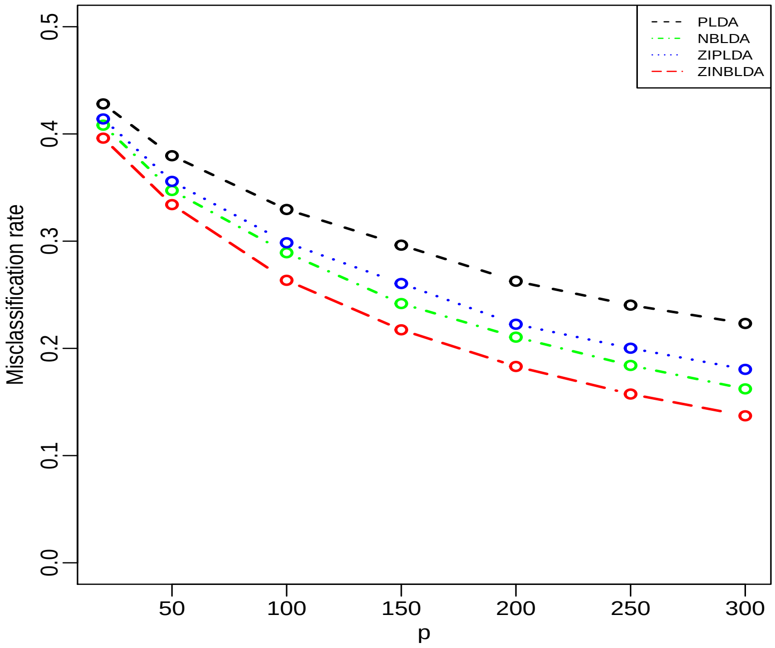

Figure S3. The misclassification rates of the four methods with different number of genes (Study 4). Here, $K = 2$, $\sigma = 0.2$, $n = 50$, $\phi = 0$, $p_0 = 0.1$ and $DE\ rate = 0.2$.

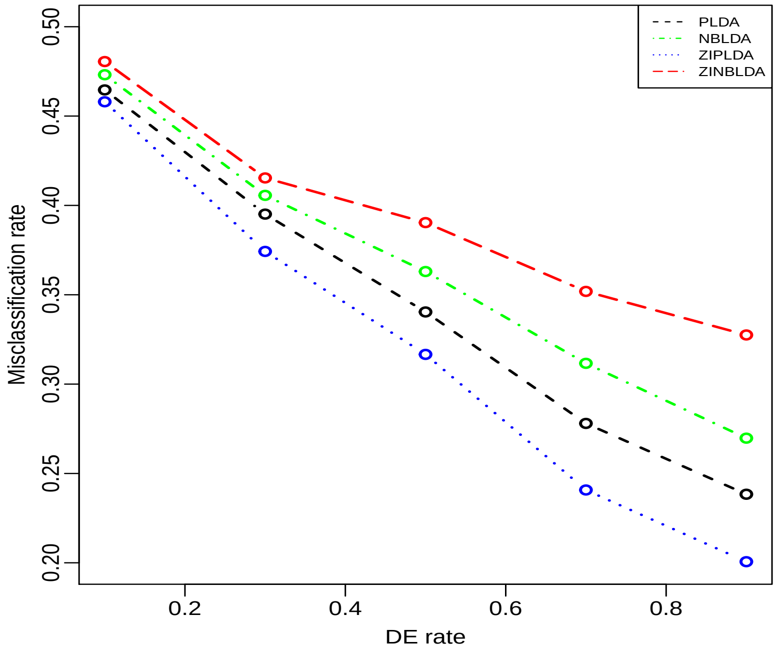

Figure S4. The misclassification rates of the four methods with different probability of differential expression genes (Study 5). Here, $K = 2$, $\sigma = 0.2$, $p = 100$, $n = 8$, $\phi = 0.2$ and $p_0 = 0.1$.

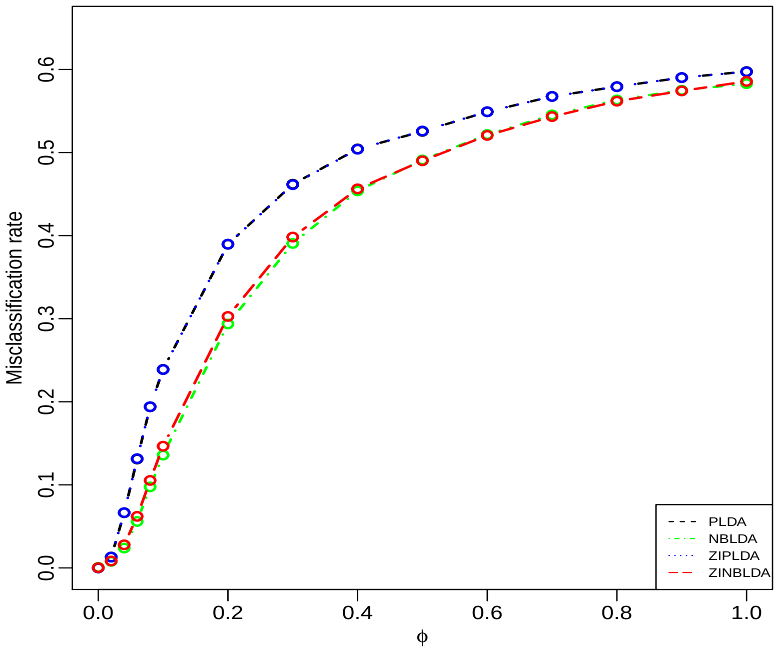

Figure S5. The misclassification rates of the four methods with different dispersions. Here, $K = 3$, $\sigma = 0.2$, $p = 100$, $n = 75$, $p_0 = 0$ and $DE\ rate = 0.2$.

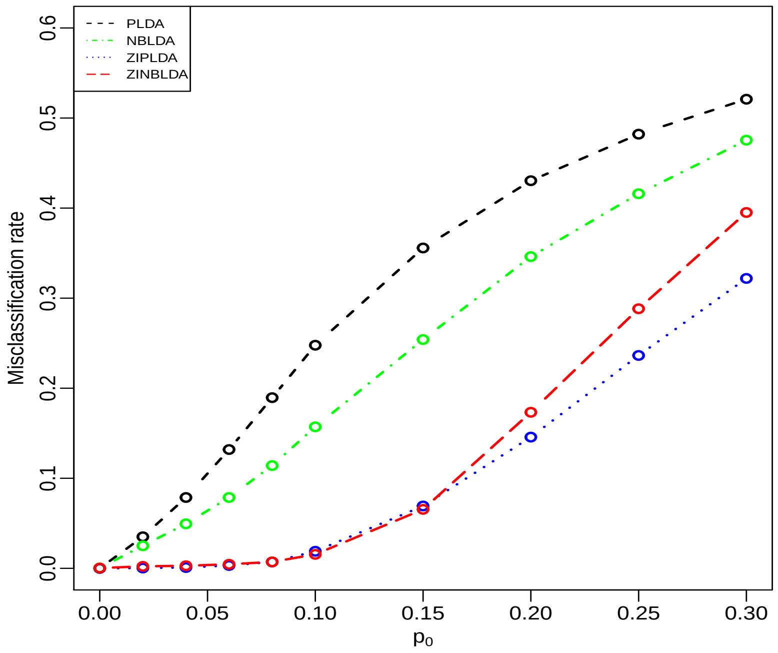

Figure S6. The misclassification rates of the four methods with different probability of excess zeros. Here, $K = 3$, $\sigma = 0.2$, $p = 100$, $n = 75$, $\phi = 0$ and $DE\ rate = 0.2$.

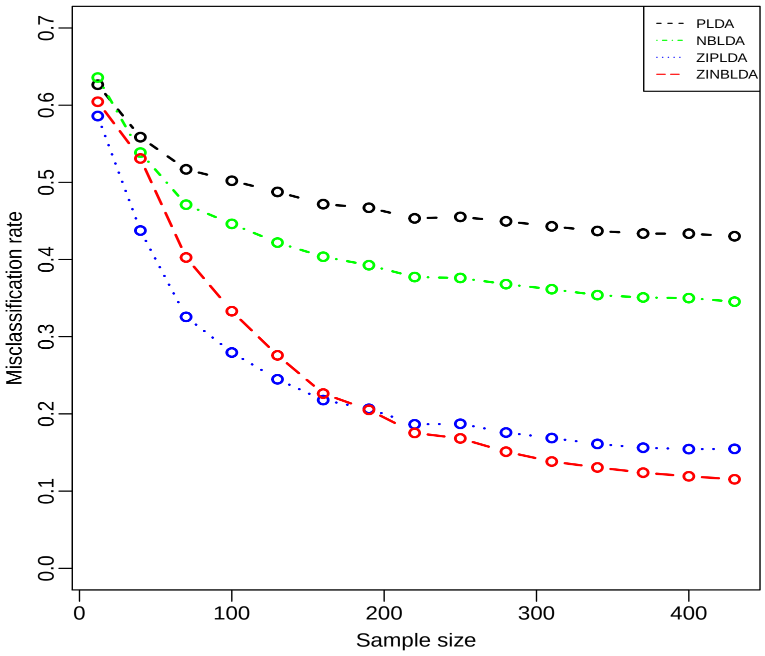

Figure S7. The misclassification rates of the four methods with different sample sizes. Here, $K = 3$, $\sigma = 0.2$, $p = 100$, $\phi = 0$, $p_0 = 0.3$ and $DE\ rate = 0.2$.

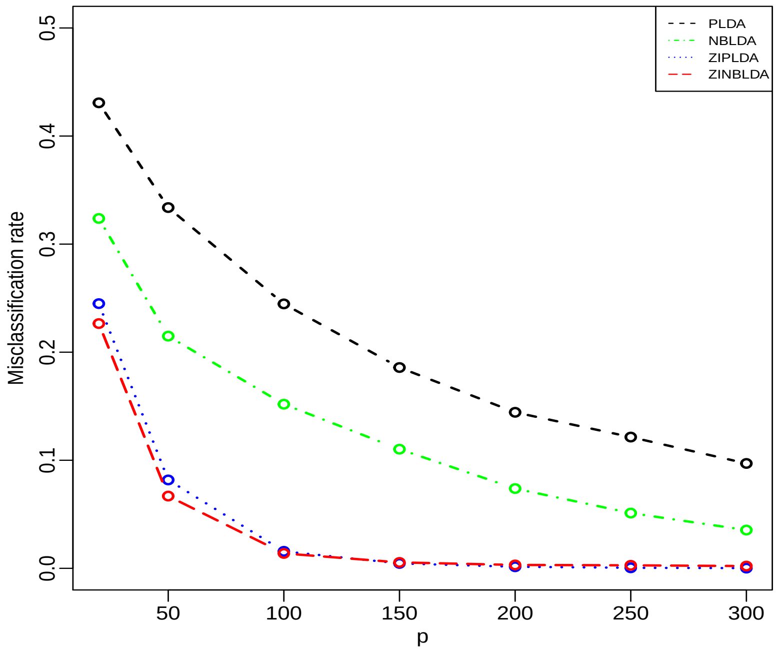

Figure S8. The misclassification rates of the four methods with different number of genes. Here, $K = 3$, $\sigma = 0.2$, $n = 50$, $\phi = 0$, $p_0 = 0.1$ and $DE\ rate = 0.2$.

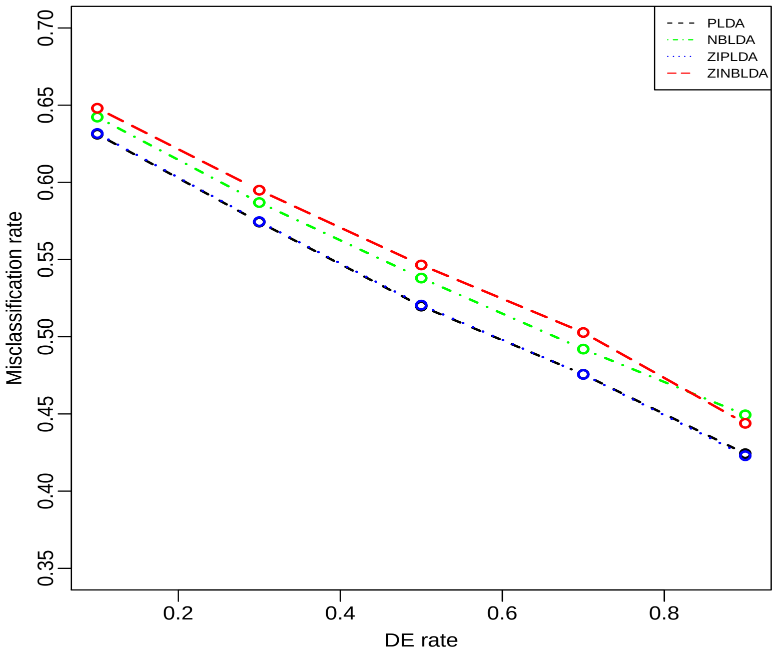

Figure S9. The misclassification rates of the four methods with different probability of differential expression genes. Here, $K = 3$, $\sigma = 0.2$, $p = 100$, $n = 12$, $\phi = 0.5$ and $p_0 = 0$.

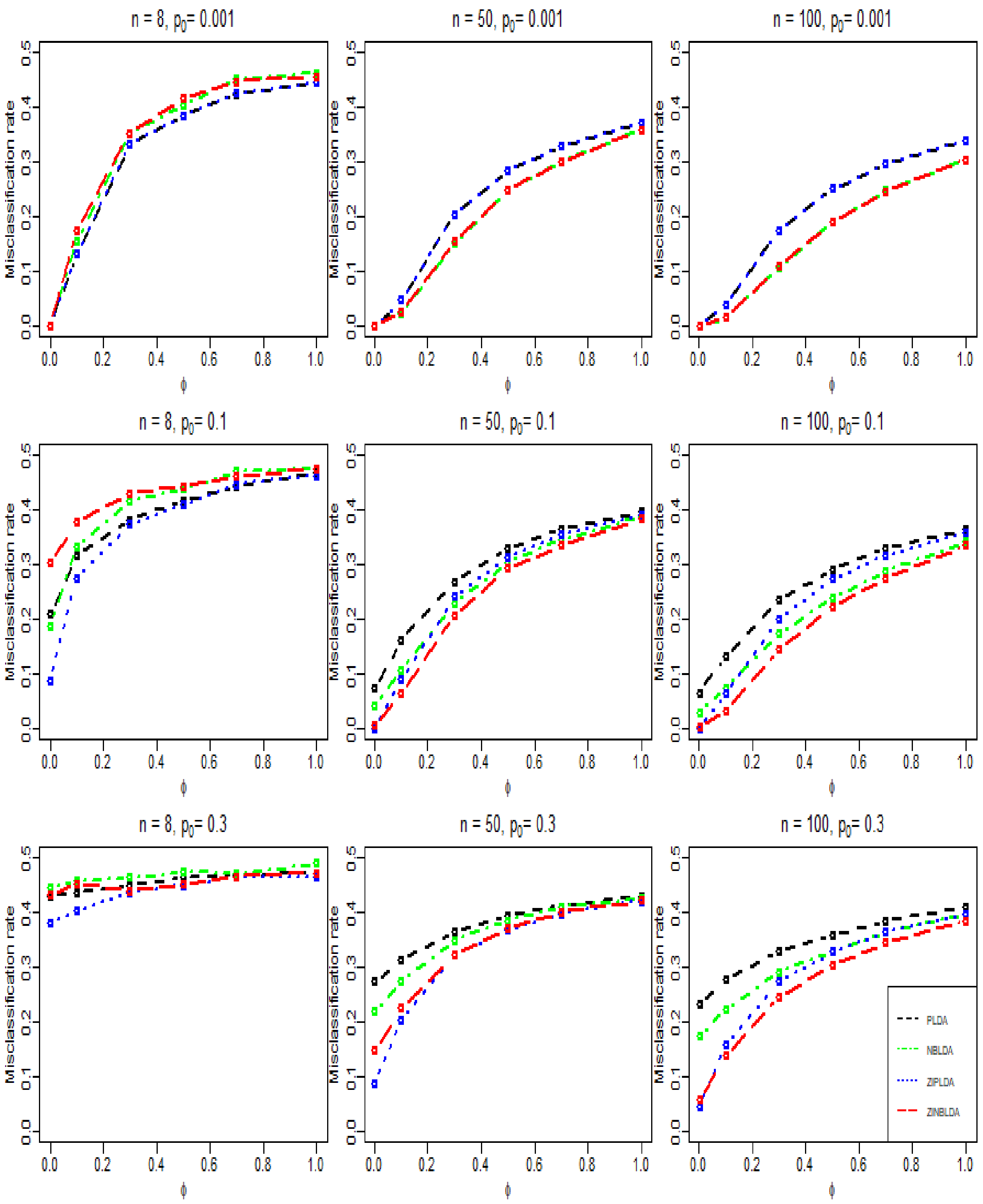

Figure S10. The misclassification rates of the four methods with different sample size, dispersions and the probability of excess zeros (Study 6). Here, $K = 2$, $\sigma = 0.2$ and $p = 100$.

Table S1. The misclassification rates of the four methods with different sample size, dispersions and the probability of excess zeros.

	ϕ	0.001	0.1	0.3	0.5	0.7	1	0.001	0.1	0.3	0.5	0.7	1	0.001	0.1	0.3	0.5	0.7	1
p_0	Methods	n=8						n=50						n=100					
	PLDA	0.00	0.13	0.33	0.38	0.42	0.44	0.00	0.05	0.20	0.28	0.33	0.37	0.00	0.04	0.17	0.25	0.30	0.34
0.001	NBLDA	0.00	0.16	0.35	0.40	0.45	0.46	0.00	0.02	0.15	0.25	0.30	0.36	0.00	0.01	0.11	0.19	0.25	0.31
	ZIPLDA	0.00	0.13	0.33	0.38	0.43	0.45	0.00	0.05	0.20	0.28	0.33	0.37	0.00	0.04	0.17	0.25	0.30	0.34
	ZINBLDA	0.00	0.17	0.35	0.42	0.45	0.46	0.00	0.03	0.15	0.25	0.30	0.36	0.00	0.02	0.11	0.19	0.25	0.30
	PLDA	0.21	0.32	0.38	0.42	0.44	0.47	0.07	0.16	0.27	0.33	0.37	0.40	0.07	0.13	0.24	0.29	0.33	0.37
0.1	NBLDA	0.19	0.33	0.42	0.44	0.47	0.48	0.04	0.11	0.23	0.30	0.35	0.39	0.03	0.08	0.17	0.24	0.29	0.34
	ZIPLDA	0.09	0.28	0.37	0.41	0.45	0.46	0.00	0.09	0.24	0.31	0.36	0.39	0.00	0.06	0.20	0.27	0.32	0.36
	ZINBLDA	0.30	0.38	0.43	0.44	0.46	0.48	0.00	0.06	0.21	0.29	0.34	0.38	0.00	0.03	0.14	0.22	0.28	0.33
	PLDA	0.43	0.44	0.45	0.46	0.47	0.47	0.27	0.31	0.37	0.39	0.41	0.43	0.23	0.28	0.33	0.36	0.38	0.41
0.3	NBLDA	0.44	0.46	0.46	0.47	0.47	0.49	0.22	0.27	0.35	0.39	0.41	0.43	0.17	0.22	0.29	0.33	0.36	0.40
	ZIPLDA	0.38	0.40	0.44	0.45	0.46	0.46	0.09	0.20	0.32	0.37	0.40	0.42	0.05	0.16	0.28	0.33	0.36	0.40
	ZINBLDA	0.43	0.45	0.44	0.45	0.47	0.47	0.15	0.23	0.32	0.37	0.40	0.42	0.06	0.14	0.25	0.30	0.34	0.38
